# Prescription patterns of somatostatin analogs in patients with acromegaly and neuroendocrine tumors

**DOI:** 10.1007/s40618-022-01875-7

**Published:** 2022-08-01

**Authors:** J. E. Machado-Alba, M. E. Machado-Duque, A. Gaviria-Mendoza, I. N. Arsof-Saab, C. A. Castellanos-Moreno, L. Botero, L. Triana

**Affiliations:** 1 Grupo de Investigación en Farmacoepidemiología y Farmacovigilancia, Universidad Tecnológica de Pereira-Audifarma S.A, Calle 105 No. 14-140, 660003 Pereira, Risaralda Colombia; 2 Ipsen Export Latin America, Mexico DF, Mexico; 3 Sanofi Colombia S. A, Bogota, Colombia; 4 Health Economics and Value Assessment, Sanofi Colombia S. A, Bogota, Colombia

**Keywords:** Acromegaly, Neuroendocrine tumors, Pharmaco-epidemiology, Somatostatin

## Abstract

**Purpose:**

Acromegaly and neuroendocrine tumors are rare diseases that, under certain conditions, can be treated with somatostatin analogs. The aim was to determine the prescription patterns of somatostatin analogs in a group of patients with acromegaly and neuroendocrine tumors affiliated with the Colombian Health System.

**Methods:**

A retrospective study. A cohort of patients from a drug dispensing database that collected all prescriptions of long-acting somatostatin analogs (octreotide, lanreotide, pasireotide). Sociodemographic variables, clinical variables (diagnosis and comorbidities) and pharmacological therapy variables (dose, changes, persistence of use, comedications) were considered.

**Results:**

A total of 213 patients were identified, including 139 (65.3%) with acromegaly and 74 (34.7%) with neuroendocrine tumors. There was a predominance of women (58.7%) and a mean age of 59.7 ± 14.5 years. The most commonly used medications were lanreotide autogel (*n* = 107; 50.2%), octreotide LAR (*n* = 102; 47.9%) and pasireotide LAR (*n* = 4; 1.9%). During follow-up, 11.3% of patients experienced modifications of therapy, with a mean duration from the beginning of treatment to the change in medication of 25 ± 15.9 months. A total of 48.9% of the patients with acromegaly and 87.1% of individuals with neuroendocrine tumors received maximum approved doses of the drug.

**Conclusion:**

Patients with acromegaly and neuroendocrine tumors in Colombia are mainly women and are most frequently treated with lanreotide autogel for acromegaly and with octreotide LAR for neuroendocrine tumors. In addition, a high proportion are managed with maximum doses of long-acting somatostatin analogs.

## Introduction

Acromegaly is a relatively rare disease with an estimated global prevalence of 40–50 cases per million inhabitants [1, 2]. It leads to alterations in patients’ quality of life and early mortality, mainly due to cardiovascular causes, and consequently generates high costs related to both medical and surgical treatments [3, 4]. The management of these patients is complex and aims to control symptoms, normalize hormone excess, and reduce tumor volume [4]. First-line treatment usually involves trans-sphenoidal adenectomy (in patients with completely resectable tumors or compromised vision), but in patients who are not eligible candidates for this procedure, those with unsuccessful pituitary surgery and those who reject the surgery [5], somatostatin analogs, such as octreotide and lanreotide, are recommended [4, 6–8]. These drugs are also used as first-line therapy in patients with un-resectable neuroendocrine tumors, which have a prevalence of less than 1%, although their incidence has increased in recent years [9]. Somatostatin analogs are also used for symptom control of metastatic carcinoid tumors, such as flushing, wheezing, telangiectasias and diarrhea [10].

Somatostatin analogs preferentially bind to somatostatin receptors SST_2_ and SST_5_, suppressing growth hormone (GH) secretion [11, 12], effectively controlling the biochemical parameters of acromegaly in 60 to 80% of patients (normal values of insulin-like growth factor 1 (IGF-1) and GH < 1 μg/L) [5]. In neuroendocrine tumors, they can lead to anti-proliferative effects by decreasing cell cycles and activating the apoptosis of cells expressing somatostatin receptors, leading to a decrease in tumor angiogenesis and growth factors expression and a reduction in the secretion of hormonal peptides [13, 14], and reduction in skin flushing and diarrhea in up to 88% of patients, as well [15]. Despite being a fairly effective therapy, the costs of this approach are significant, and in some cases and some countries, it is difficult or impossible to access to these medications, leading doctors to use alternative treatments [3, 16, 17].

In a report of patients in Colombia with acromegaly and neuroendocrine tumors (between 2011 and 2015), the use of long-acting somatostatin analogs was described. Octreotide was the most frequently used (in 56.1% of the cases), followed by lanreotide (43.9%). In addition, 4.5% of patients changed from one medication to another [17]. Furthermore, in 2013, pasireotide LAR became available in the public health system formulary. It is another somatostatin analog also administered every 28 days and approved for patients with acromegaly [18, 19].

Since acromegaly is a condition hidden by many comorbid disorders, its diagnosis is usually delayed for several years leading to increased mortality versus the general population [20, 21]. Similarly, gastroenteropancreatic neuroendocrine tumors are likely to be diagnosed in advanced stage due to many gastrointestinal disorders masking the neuroendocrine tumor and increasing the complexity and the cost of the treatment. [9, 10, 22, 23]. Therefore, the management of these conditions in the real-life setting is a topic of interest. The Colombian Health System offers universal coverage to the entire population through two regimes: the contributory scheme, which is paid by workers and employers, and another scheme that is subsidized by the state. It also has a benefit plan (PBS) that includes some medications used for the treatment of acromegaly and neuroendocrine tumors. Thus, it was proposed to determine the prescription and use patterns of somatostatin analogs in a group of patients with acromegaly and neuroendocrine tumors affiliated with the Colombian Health System between 2015 and 2020.

## Methods

A retrospective study of a cohort of patients was conducted aiming to describe the prescription pattern of long-acting somatostatin analogs (octreotide LAR, lanreotide autogel and pasireotide LAR) and their dispensation trend over time from 2015 to 2020. From a population of 6.5 million people affiliated with the contributory and subsidized regimes of the Colombian Health System of different health insurance entities, patients with prescriptions for somatostatin analogs were identified, and a monthly follow-up was performed with the objective of evaluating changes in therapy.

The study database, validated and reviewed by a pharmacologist, was designed to allow the collection of groups of patient variables during the observation period. These variables are described below:

1. Sociodemographic variables: Age, sex and affiliation regime (contributory or subsidized).
2. Clinical variables: Clinical diagnosis identified in the prescription (differentiating between patients with acromegaly or neuroendocrine tumor), modifications (dose or medication) and persistence of treatment use, determined by the number of dispensations of drugs (persistence during the first 12 months of use).
3. Use of somatostatin analogs (patterns and dispensation trend): Number of patients with a monthly prescription during the follow-up period (trend), specialty of the prescribing physician and the prescribed dose. To quantify the amount dispensed, the defined daily dose (DDD) recommended by the World Health Organization (WHO) as the international standard for pharmaco-epidemiological studies was used as the technical unit of measurement.
4. The following comorbidities and their respective medications were considered: (a) diabetes mellitus; (b) Parkinson's disease; (c) HIV-AIDS; (d) depressive disorder; (e) chronic obstructive pulmonary disease; (f) dyslipidemia; (g) hypothyroidism; (h) ischemic heart disease; (i) arterial hypertension, (j) hyperprolactinemia, (k) cardiac arrhythmia, and (l) rheumatoid arthritis.

For the analysis of the data, the statistical package SPSS Statistics, version 26.0 (IBM, USA) for Windows was used. Means with standard deviation and frequency with proportions were calculated. The sociodemographic and usage variables, including changes in therapy and co-medication, were reported for the total population of somatostatin analog users (patterns and trend of use). The persistence of use (number of months in which treatment was dispensed during the first year of follow-up), the total duration of use and the changes in therapy throughout the follow-up period, including the time (in months) until the first change in therapy, were identified.

DDD for the study molecules were the following: 3 mg/day for lanreotide (90 mg/month), 0.7 mg/day for octreotide (21 mg/month) and 1.2 mg/day for pasireotide (36 mg/month), according to the Anatomical Therapeutic Chemical (ATC) classification of the WHO. The ratio of the average dose administered to the patients to the monthly DDD (average dose/DDD) was calculated. Similarly, the proportions of patients with maximum doses and with doses higher than the maximum were calculated for each medication. The maximum doses were octreotide 30 mg/month, lanreotide 120 mg/month and pasireotide 60 mg/month.

The protocol was approved by the Bioethics Committee of the *Universidad Tecnológica de Pereira* in the category of risk-free research according to resolution 8430/1993 of the Ministry of Health of Colombia, and the principles established by the Declaration of Helsinki were respected (Endorsement Code: 20–150,321).

## Results

A total of 213 patients who used long-acting somatostatin analogs were included in the analysis. Of these, 139 were diagnosed with acromegaly (65.3%), and 74 were diagnosed with neuroendocrine tumors (34.7%). There was a predominance of females (*n* = 125; 58.7%) and an average age of 59.7 ± 14.5 years for the total included population. A total of 72.3% (*n* = 154) participated in the contributory regime of the health system. Table 1 shows the sociodemographic characteristics, comorbidities and concomitant therapies of each group of patients.

**Table 1:** Sociodemographic characteristics, comorbidities and concomitant therapies for a group of patients diagnosed with acromegaly and neuroendocrine tumors and treated with somatostatin analogs who were affiliated with the Colombian Health System, 2015–2020

Sociodemographic	Acromegaly								Neuroendocrine tumors					
Variables	*N* = 139	%	Ocreotide	*n* = 27	Lanreotide	*n* = 78	Pasireotide	*n* = 4	*N* = 74	%	Ocreotide	*n* = 45	Lanreotide	*n* = 29
Age (mean ± standard deviation)	58.4 ± 14.1		61.1 ± 13.7		57.6 ± 13.8		37.8 ± 4.5		62.4 ± 15.2		62.6 ± 14.5		62.0 ± 16.7	
Female sex	82	59.0	34.0	59.6	45.0	57.7	3.0	75.0	43	58.1	25.0	55.6	18.0	62.1
Comorbidities														
Arterial hypertension	75	54.0	32.0	56.1	42.0	53.8	1.0	25.0	30	40.5	18.0	40.0	12.0	41.4
Diabetes Mellitus	50	36.0	24.0	42.1	25.0	32.1	1.0	25.0	16	21.6	10.0	22.2	6.0	20.7
Hypothyroidism	55	39.6	17.0	29.8	20.0	25.6	0.0	0.0	13	17.6	10.0	22.2	3.0	10.3
Hyperprolactinemia	26	18.7	12.0	21.1	14.0	17.9	0.0	0.0	1	1.4	1.0	2.2	0.0	0.0
Dyslipidemia	18	12.9	9.0	15.8	9.0	11.5	0.0	0.0	6	8.1	3.0	6.7	3.0	10.3
Chronic obstructive pulmonary disease	1	7.0	0.0	0.0	1.0	1.3	0.0	0.0	6	8.1	5.0	11.1	1.0	3.4
Ischemic heart disease	7	5.0	5.0	8.8	2.0	2.6	0.0	0.0	1	1.4	1.0	2.2	0.0	0.0
Rheumatoid arthritis	7	5.0	3.0	5.3	4.0	5.1	0.0	0.0	4	5.4	3.0	6.7	1.0	3.4
Depressive disorder	7	5.0	3.0	5.3	4.0	5.1	0.0	0.0	8	10.8	5.0	11.1	3.0	10.3
Cardiac arrhythmia	3	2.2	2.0	3.5	1.0	1.3	0.0	0.0	4	5.4	3.0	6.7	1.0	3.4
HIV	2	1.4	2.0	3.5	0.0	0.0	0.0	0.0	1	1.4	0.0	0.0	1.0	3.4
Parkinson's disease	1	0.7	1.0	1.8	0.0	0.0	0.0	0.0	1	1.4	1.0	2.2	0.0	0.0
Complementary medications														
Cabergoline	48	34.5	19.0	33.3	28.0	35.9	1.0	25.0	0	0	0.0	0.0	0.0	0.0
Bromocriptine	13	9.4	10.0	17.5	3.0	3.8	0.0	0.0	0	0	0.0	0.0	0.0	0.0
Everolimus	1	0.7	1.0	1.8	0.0	0.0	0.0	0.0	17	23	9.0	20.0	8.0	27.6
Pegvisomant	2	1.4	1.0	1.8	1.0	1.3	0.0	0.0	0	0	0.0	0.0	0.0	0.0
Sunitinib	0	0.0	0.0	0.0	0.0	0.0	0.0	0.0	2	2.7	1.0	2.2	1.0	3.4
Short-acting octreotide	0	0.0	0.0	0.0	0.0	0.0	0.0	0.0	5	6.8	1.0	2.2	4.0	13.8
Analgesics/other														
Acetaminophen	84	60.4	38.0	66.7	43.0	55.1	3.0	75.0	46	62.2	29.0	64.4	17.0	58.6
Nonsteroidal anti-inflammatory drugs	68	48.9	28.0	49.1	38.0	48.7	2.0	50.0	32	43.2	20.0	44.4	12.0	41.4
Proton pump inhibitor	71	51.1	32.0	56.1	36.0	46.2	3.0	75.0	45	60.8	29.0	64.4	16.0	55.2
Bronchodilators	24	17.3	11.0	19.3	11.0	14.1	2.0	50.0	18	24.3	12.0	26.7	6.0	20.7
Endocrine														
Systemic corticosteroids	56	40.3	25.0	43.9	29.0	37.2	2.0	50.0	26	35.1	18.0	40.0	8.0	27.6
Levothyroxine	55	39.6	22.0	38.6	31.0	39.7	2.0	50.0	14	18.9	11.0	24.4	3.0	10.3
Oral antidiabetics	49	35.3	25.0	43.9	23.0	29.5	1.0	25.0	12	16.2	7.0	15.6	5.0	17.2
Insulins	24	18.0	10.0	17.5	15.0	19.2	0.0	0.0	9	12.2	5.0	11.1	4.0	13.8
Cardiovascular														
Antihypertensives	69	49.6	32.0	56.1	37.0	47.4	0.0	0.0	30	40.5	20.0	44.4	10.0	34.5
Statins	62	44.6	30.0	52.6	31.0	39.7	1.0	25.0	18	24.3	12.0	26.7	6.0	20.7
Diuretics	45	32.4	23.0	40.4	22.0	28.2	0.0	0.0	20	27	12.0	26.7	8.0	27.6
Aspirin	32	23.0	13.0	22.8	19.0	24.4	0.0	0.0	13	17.6	9.0	20.0	4.0	13.8
Platelet antiaggregants	5	3.6	3.0	5.3	2.0	2.6	0.0	0.0	1	1.4	0.0	0.0	1.0	3.4
Psychopharmaceuticals														
Opioids	60	43.2	28.0	49.1	30.0	38.5	2.0	50.0	43	58.1	29.0	64.4	14.0	48.3
Anticonvulsants	27	19.4	11.0	19.3	15.0	19.2	1.0	25.0	19	25.7	14.0	31.1	5.0	17.2
Antidepressants	29	20.9	11.0	19.3	17.0	21.8	1.0	25.0	18	37.8	17.0	37.8	11.0	37.9
Benzodiazepines	10	7.2	7.0	12.3	3.0	3.8	0.0	0.0	10	13.5	9.0	20.0	1.0	3.4
Antipsychotics	5	3.6	2.0	3.5	3.0	3.8	0.0	0.0	11	14.9	8.0	17.8	3.0	10.3
Antiparkinsonians	1	0.7	1.0	1.8	0.0	0.0	0.0	0.0	1	1.4	0.0	0.0	1.0	3.4

The general frequencies of use of the medications were as follows: lanreotide autogel (*n* = 107; 50.2%), octreotide LAR (*n* = 102; 47.9%) and pasireotide LAR (*n* = 4; 1.9%). Among the patients with acromegaly, the most commonly used complementary therapy was cabergoline (*n* = 48; 34.5%), while among those with neuroendocrine tumors, it was everolimus (*n* = 17; 23.0%). Table 2 shows the patterns of somatostatin analog use according to the disease.

**Table 2:** Patterns of somatostatin analog use in a group of patients with acromegaly and neuroendocrine tumors who were affiliated with the Colombian health system, 2015–2020

Variable	*n*	%	Women (%)	Mean dose-mg/month (SD)	nDDD	Ratio of the mean dose over maximum dose of the drug	Proportion of patients with maximum dose* *n* (%)	Proportion of patients above the maximum dose *-*n* (%)	Mean duration-first use (months)	Mean duration-second use (months)	Number of dispensations- first 12 months (mean; SD)	Total usage time (months)
Acromegaly (*n* = 139)												
Lanreotide autogel	78	56.1	57.7%	104.6 (39.8)	1.16	0.86	38 (48.7)	2 (2.6)	3.2	3.1	7.0 ± 3.4	25
Octreotide LAR	57	41.0	59.6%	27.0 (11.3)	1.29	0.90	28 (49.1)	5 (8.8)	3.2	3.1	6.7 ± 3.1	25
Pasireotide LAR	4	2.9	75.0%	35.0 (10.0)	0.97	0.58	0 (0.0)	0 (0.0)	2.5	4.3	5.7 ± 4.2	15
Neuroendocrine tumors (*n* = 74)												
Octreotide LAR	45	60.8	55.6%	32.4 (16.0)	1.54	1.06	38 (84.4)	3 (6.7)	2.5	3.1	5.1 ± 3.4	29
Lanreotide autogel	29	39.2	62.1%	117.9 (17.8)	1.31	0.98	26 (89.7)	1 (3.4)	2.1	1.8	4.3 ± 3.5	10

*SD* standard deviation, *nDDD* ratio between the mean dose and the defined daily dose

^*^Maximum monthly dose: octreotide LAR 30 mg, lanreotide autogel 120 mg, pasireotide LAR 60 mg

Among the patients with acromegaly, 19 of those who used octreotide LAR (33.3%), 28 of those who used lanreotide autogel (35.9%) and one patient who used pasireotide LAR (25.0%) were treated concomitantly with cabergoline during the study period. Nine (20.0%) patients with neuroendocrine tumors who were treated with octreotide LAR and eight (27.6%) of those who used lanreotide autogel were concomitantly treated with everolimus. Of the patients with neuroendocrine tumors, only one who was treated with octreotide LAR (2.2%) and one who was treated with lanreotide autogel (3.4%) used sunitinib.

Among the patients with neuroendocrine tumors, the mean dose of octreotide LAR was higher than the maximum approved and recommended dose of 30 mg, while the mean dose of lanreotide autogel was lower than the maximum approved and recommended dose of 120 mg, as shown in Table 2, which presents the ratio of the mean dose to the maximum dose of the drugs.

During the follow-up period, 24 patients underwent treatment modifications (11.3%), with a mean duration of 25 ± 15.9 months from the beginning of treatment until the change in therapy. Modifications were more frequently observed in patients with acromegaly treated with octreotide LAR (14% switched to lanreotide autogel). Among those with neuroendocrine tumors, there were more patients who switched from octreotide to lanreotide (8.8%) than those who switched from lanreotide to octreotide (3.5%). Table 2 also shows the mean number of dispensations in the first year of use (persistence) and Table 3 shows the changes in the studied drugs according to the diagnosis.

**Table 3:** Changes in treatment with somatostatin analogs in a group of patients diagnosed with acromegaly or neuroendocrine tumors who were affiliated with the Colombian Health System from 2015 to 2020

Variable	*n* (%)	No change of medication *n* (%)	Switch to lanreotide autogel *n* (%)	Switch to octreotide LAR *n* (%)	Switch to pasireotide LAR *n* (%)	Time in months to change of therapy (mean; SD)
Acromegaly (*n* = 139)						
Lanreotide autogel	78 (56.1)	69 (88.4)	NA	4 (5.1)	5 (6.4)	27.1 (15.6)
Octreotide LAR	57 (41.0)	47 (82.5)	8 (14.0)	NA	2 (3.5)	17.4 (27.6)
Pasireotide LAR	4 (2.9)	4 (100.0)	NA	NA	NA	NA
Neuroendocrine tumors (*n* = 74)						
Octreotide LAR	45 (60.8)	41 (91.1)	4 (8.8)	NA	NA	17.4 (14.5)
Lanreotide autogel	29 (39.2)	28 (96.5)	NA	1 (3.5)	NA	10.2 (NA)

*SD* standard deviation

## Discussion

From the results obtained, it was possible to identify patients diagnosed with acromegaly and neuroendocrine tumors who were managed with long-acting somatostatin analogs during the five years of follow-up and to determine sociodemographic variables, comorbidities, concomitant therapies and treatment patterns associated with these medications. This information provides greater knowledge about pharmacological therapy based on real-life data that can be useful to improve access and care programs [2, 24].

The literature reports that most acromegaly patients are diagnosed at 40–45 years old [20]. The mean age in this study was 58 years, a fact that may be explained by the later diagnosis of this condition in Colombia than in developed countries and because patients who use long-acting somatostatin analogs usually have an evolution of the disease longer than 10 years (time between symptoms onset and the final diagnosis) [25]. In addition, there was a predominance of female patients among those diagnosed with acromegaly, similar to the findings of a previous study conducted in Colombia [17]. Among the patients diagnosed with neuroendocrine tumors, the mean age was similar to that reported in the literature, in which the median age at the diagnosis for a wide variety of gastroenteropancreatic neuroendocrine tumors is approximately 63 years, with slight variations according to the location of the tumor [23, 26, 27], in addition, it is clear to note that the frequency of neuroendocrine tumors treated with somatostatin analogs registered in the database is low, which can be explained because in many patients the ICD 10 diagnosis is not registered in the database or they use other therapies to control the disease.

The identification of comorbidities in the patients with acromegaly showed a high frequency of associated cardiovascular and metabolic disease [20], including a high prevalence of arterial hypertension, diabetes mellitus and dyslipidemia, conditions that increase the risk of cardiovascular events [28, 29]. This finding implies that the medical team should be aware of these possible comorbidities and actively search for them to provide timely treatment that can impact cardiovascular outcomes; additionally, acromegaly itself is associated with arterial hypertension, diabetes mellitus and cardiomyopathies [20, 29]. Furthermore, it should be noted that treatment with somatostatin analogs, especially pasireotide LAR, has been associated with hyperglycemia [30, 31]. Similarly, among the patients with neuroendocrine tumors, it was determined that main comorbidities were arterial hypertension and diabetes mellitus, as has been reported in epidemiological studies that identified these diagnoses and obesity as the main comorbidities of neuroendocrine tumors [10, 15]. More than 10% of the patients of this cohort were diagnosed with a depressive disorder. Such psychiatric comorbidity has been previously described and associated with the pathophysiology of neuroendocrine tumors [32] and may be a subject to explore in future studies and to be considered as part of holistic management of these patients. It is also noteworthy that the main concomitant medication in the management of acromegaly was cabergoline, a therapy that was only included in the PBS of the Colombian Health System until 2021, so patients required an extra approval to access this therapy, which explains why up to almost 10% received bromocriptine than if they were in the PBS and it was easier to access [33].

Analysis of long-acting somatostatin analogs used by patients with acromegaly showed a predominance of lanreotide autogel, which was used by more than half of the patients, followed by octreotide LAR and a small proportion of pasireotide LAR prescriptions in doses close to those recommended. However, approximately half of the users of octreotide LAR and lanreotide autogel were receiving the maximum doses, a situation similar to that reported in a similar study conducted in Colombia in 2017, which also found that octreotide LAR was used at higher doses than lanreotide autogel [17]. In addition, average doses that are higher than the maximum dose were identified in this study among patients with acromegaly being treated with octreotide LAR and among patients with neuroendocrine tumors; these high doses can lead to an increase in the associated treatment costs.

Another noteworthy point regarding somatostatin analogs is their persistence of use, because they are highly effective at normalizing GH concentrations in up to 70% of patients and IGF-1 in up to 80% [34, 35]. For many patients, prescription should be maintained for the long term or even indefinitely, since these drugs suppress GH secretion and their discontinuation can result in an adverse increase in GH [36]; an exception is in patients with a good response, in whom the doses can be spaced apart [37]. The present analysis identified that during the observation period, octreotide LAR and lanreotide autogel treatments were dispensed 5.7 and 7 times in the first year of treatment, respectively, for acromegaly patients; this suggests that the patients experienced interruptions in treatment, considering that these therapies should be administered on a monthly basis to achieve and maintain a biochemical and clinical control, at least at the beginning of treatment. This scenario was previously reported by [16] in a study of 1308 patients with acromegaly in the United States [16]. This situation could also impact patients’ quality of life, the control of the disease and its complications and the safety of the therapy [38]. These findings suggest the need for new studies that identify the factors associated with a lack of persistence in or adherence to the use of these drugs; examine the differences in effectiveness, real-world safety, impact on quality of life and control of comorbidities; and explore difficulties with access and delays in health systems’ administrative processes.

Regarding the use of somatostatin analogs among patients diagnosed with neuroendocrine tumors, octreotide LAR was used for a much longer time than lanreotide autogel. The most logical explanation for this finding is the continuity of therapy with octreotide LAR from monotherapy to combined therapy with everolimus or radionuclides once patients exhibit disease progression based on the evidence from the RADIANT-1, RADIANT-2 and NETTER-1 studies; however, these data require further investigation [37, 38].

Regarding the doses used by patients with neuroendocrine tumors, a higher mean monthly dose was identified for octreotide LAR compared to that of lanreotide autogel (32.4 mg vs. 117.9 mg, respectively) relative to the recommended doses (30 mg for octreotide and 120 mg for lanreotide). Additionally, twice as many patients received doses above the maximum for octreotide LAR than for lanreotide autogel. This finding of octreotide LAR prescriptions that exceeded the maximum dose of 30 mg every 28 days, particularly for patients with neuroendocrine tumors, coincides with the reports of similar studies in Europe and the United States. For example, the study by Fagnani F et al. evaluated the doses of lanreotide autogel and octreotide LAR recorded in the database of the national health system of France (which included 99% of the total population of the country) and reported the same finding as the present study, namely, that the proportion of patients with neuroendocrine tumors who were treated at doses higher than the maximum approved among those treated with octreotide LAR (11.2%, *n* = 100) doubled the proportion of patients treated with doses above the maximum recommended for lanreotide autogel (5.5%, *n* = 83)[11]. In the present study, these higher doses used in patients with neuroendocrine tumors was also noted when comparing the proportions of patients with maximum doses (above 80%) against those in the group of patients with acromegaly (below 50%).

A slightly higher number of dispensations for octreotide LAR than for lanreotide autogel was identified; however, this was still well below the expected number of dispensations (1 per month = 12 per year), a finding to be further investigated in future studies.

In determining the impact of this study, some important limitations should be taken into account, such as the small number of patients included, which is related to the low prevalence of acromegaly and neuroendocrine tumors, and the lack of such clinical data as the severity of the disease, the location and type of neuroendocrine tumors, the history of surgical management and the reasons for therapy change or discontinuation. Detailed information on the prescription was not available to accurately determine the dosing interval, thus the dose/month was calculated from monthly drug deliveries on usual dosing basis every 4 weeks. All this information could be useful to explain some of the findings related in the present study. For this same reason, conclusions regarding adherence and persistence may have limited comparability among patients.

Based on above findings, it can be concluded that patients with acromegaly and neuroendocrine tumors in Colombia are primarily women who receive lanreotide autogel more frequently for the treatment of acromegaly and octreotide LAR more frequently for neuroendocrine tumors. In addition, approximately half of patients with acromegaly and more than 80% of those with neuroendocrine tumors receive the maximum doses of the different somatostatin analogs, with a low proportion of switching between drugs and intermediate persistence of use throughout the first year of treatment. These results can be useful for physicians in the areas of neurosurgery, endocrinology, and oncology and for decision-makers, as well, to improve treatment adherence, medication use and the clinical outcomes in these patients.

## Data Availability

protocols.io. dx.doi.org/10.17504/protocols.io.n92ldz5xov5b/v1.
